# COPe-support - a multi-component digital intervention for family carers for people affected by psychosis: study protocol for a randomized controlled trial

**DOI:** 10.1186/s12888-020-02528-w

**Published:** 2020-03-17

**Authors:** Jacqueline Sin, Claire Henderson, Victoria Cornelius, Tao Chen, Jack Elkes, Luke A. Woodham, Aurora Sesé Hernández, Dominique Spence-Polin, Rachel Batchelor, Steve Gillard

**Affiliations:** 1 Population Health Research Institute, St George’s, University of London, Cranmer Terrace, London, SW17 0RE England, UK; 2 School of Psychology & Clinical Language Sciences, University of Reading, Earley Gate, Reading, RG6 6AL England, UK; 3 Health Service & Population Research Department, King’s College London, Institute of Psychiatry, Psychology & Neuroscience, De Crespigny Park, London, SE5 8AF England, UK; 4 Imperial Clinical Trials Unit, School of Public Health, Imperial College London, Stadium House, 68 Wood Lane, London, England W12 7RH UK; 5 Department of Clinical Sciences, Liverpool School of Tropical Medicine, Pembroke Place, Liverpool, L3 5QA England, UK; 6 Institute of Medical and Biomedical Education, St George’s, University of London, Cranmer Terrace, London, SW17 0RE England, UK

**Keywords:** eHealth, eMentalHealth, Digital interventions, Carers, Psychosis, RCT, Wellbeing

## Abstract

**Background:**

Psychosis often causes significant distress and impacts not only in the individuals, but also those close to them. Many relatives and friends (‘carers’) provide long-term support and need resources to assist them. We have co-produced a digital mental health intervention called COPe-support (Carers fOr People with Psychosis e-support) to provide carers with flexible access to high quality psychoeducation and interactive support from experts and peers. This study evaluates the effectiveness of COPe-support to promote mental wellbeing and caregiving experiences in carers.

**Methods:**

This study is a single-blind, parallel arm, individually randomized controlled trial (RCT) comparing COPe-support, with attention control. Both groups continue to receive usual care. COPe-support provides interactive web-based psychoeducation on psychosis-related issues, wellbeing-promotion and network support through forums. The attention-control is a non-interactive online information resource pack. Carers living in England are eligible if they provide at least weekly support to a family member or close friend affected by psychosis, and use internet communication (including emails) daily. All trial procedures are run online, including collection of outcome measurements which participants will directly input into our secure platform. Following baseline assessment, a web-based randomization system will be used to allocate 360 carers to either arm. Participants have unlimited access to the allocated condition for 40 weeks. Data collection is at three time points (10, 20, and 40 weeks after randomization). Analyses will be conducted by trial statisticians blinded to allocation. The primary outcome is mental wellbeing measured by Warwick Edinburgh Mental Wellbeing Scale (WEMWBS), at 20 weeks. As well as an intention-to-treat analysis, a complier average causal effect (CACE) analysis will be conducted to estimate the intervention effect in participants who have accessed COPe-support content twice or more. The secondary objectives and analysis will examine other health and caregiving-related outcomes and explore mechanisms. In a process evaluation, we will interview 20% of the intervention arm participants regarding the acceptability of COPe-support. We will explore in detail participants’ usage patterns.

**Discussion:**

The results of this trial will provide valuable information about the effectiveness of COPe-support in promoting wellbeing and caregiving experiences in carers.

**Trial registration:**

The RCT is registered with the Current Controlled Trials registration (ISRCTN 89563420, registration date: 02/03/2018).

## Background

World-wide, an ever-increasing population is living longer in the community despite long-term illness. In addition to the advances of health care, one crucial element that support and sustain this population in their own home is the unpaid care and support provided by relatives and friends [1]. In the UK, it is estimated that approximately 1.5 million people are caring for a family member or friend with a mental illness [2–4]. Of these illnesses, psychosis is recognised as among the most common and severe [2, 3]. Often, people with psychosis require long-term treatment and support across a range of life domains, including emotional support, financial and practical assistance for daily living activities [5, 6]. Therefore, the importance of relatives and friends - referred to carers hereafter - is well established. Extant research evidence has established that individuals who receive support and care from their familial networks have a better prognosis and enhanced quality of life [3, 7, 8]. Conversely, the responsibility of caring often means that carers of people with psychosis experience high levels of distress and increased vulnerability to ill health [9–12]. Furthermore, carers’ psychiatric morbidities are shown to be inversely correlated with the amount of care they provide; that is, the higher amount of care provided leading to poorer mental health [13]. Poor mental health in carers can further negatively impact their caregiving capacity [9, 14]. Carers who are unwell themselves or feel they lack resources to cope are less likely to engage in caring for their loved ones in a positive manner [6].

Consequently, a body of research has been undertaken to establish psychosocial interventions which can best support carers [5, 6, 15]. Among these, psychoeducation (i.e. information-giving on the illness condition and related caregiving and problem-solving strategies) especially those integrated with a peer support element between carers in a group setting, has the strongest evidence base for its effectiveness in enhancing carers’ knowledge and coping with their caring roles [2, 16]. Carers also identify that sharing mutual support and learning with other carers as particularly useful in reducing their sense of isolation [5, 16]. In the UK, psychoeducation is recommended by the National Institute for Health and Care Excellence (NICE) guidelines on psychosis and schizophrenia for all carers [2].

With digital health interventions becoming more widespread and popular across a range of public mental health topics in the recent time, interventions targeting carers supporting a loved one with long-term illness have increasingly adopted an online medium [15, 17]. Many carers lead a busy lifestyle with multiple commitments in addition to caregiving. Digital interventions are potentially flexible, accessible and yet adaptable to individualized needs and schedule. Such interventions, using an enriched and dynamic online environment, are particularly suited to delivering psychoeducation, wellbeing promotion strategies, and network support with healthcare professionals and peers [18–20].

Emerging research provides increasing evidence to promote digital mental health (or e-mental health) as an effective means to disseminate interventions to carers [15]. Across a wide range of long term illnesses, there is a particularly strong evidence base for psychoeducational interventions treating mental health morbidities in carers of people with dementia, stroke, and cardiovascular diseases. In contrast, both the volume of literature and research evidence are less established in mental health arena, with the exception of eating disorders [15, 19, 21]. Few such interventions are available for psychosis carers and even fewer have been trialled rigorously [17]. Hence, the EFFIP (E-support for Families and Friends of Individuals affected by Psychosis) Project was established to fill these research gaps to develop and evaluate a digital health intervention for carers supporting a relative or close friend with psychosis [22]. The EFFIP project is a five-year research programme encompassing theoretical development work and participatory research to develop the digital health intervention which is then evaluated for effectiveness in promoting carers’ wellbeing [5, 6, 17]. The participatory study through which the intervention, called “COPe-support (Carers fOr People with Psychosis e-support)” was co-produced, is published elsewhere [22]. COPe-support provides high quality psychoeducation and interactive support to carers entirely through the internet, promoting flexible access and individualized choice of content, pace, and engagement for its users [22]. It is expected that, if effective, COPe-support can help overcome some of the barriers associated with implementing evidence-based psychosocial interventions. These include lack of resources to prioritise carers’ needs on a par with those they care for, stigma, accessibility, and the mis-match between the conventional face-to-face service provision and the diverse needs of carers [6, 17, 22].

There is a strong argument that interventions delivered online should also be evaluated online to maximize the trial’s external validity and some aspects of internal validity [23]. Firstly, it is of interest to establish whether the profile of carers using a digital intervention is representative of the wider populations, including those using conventional services (such as face-to-face carers groups, individual psychological intervention). Secondly, as COPe-support is delivered entirely online and requires the carers to self-pace and self-tailor the multiple components suiting their own needs and schedule, it is only logical to collect evaluation data directly from them using an online medium. Thirdly, while arguably the web-based media offers a low-cost platform through which the intervention can be delivered to a critical mass with a high level of fidelity to its design, research on online trials show that they can be inferior to conventional trials in terms of adherence and retention rates [17, 23–25]. It is important for both the trial and the facilitation of COPe-support to consider effective strategies to engage with carers in line with its online delivery. Finally, as digital interventions allow their users a self-directed approach in using the content, intervention usage or adherence is often very heterogenous. It is important to explore the differential benefits of the intervention use in participants with different usage patterns [26–28].

The proposed COPe-support trial aims to evaluate the clinical effectiveness of COPe-support in promoting mental wellbeing and caregiving experiences in relatives and close friends who support a loved one affected by psychosis.

### Study aims and objectives

This trial aims to determine the effectiveness of the COPe-support intervention and usual care (UC), compared with a non-interactive web-based information resource (which is also provided within COPe-support) and usual care. The comparator is an attention-matched control while also representing the current online resources widely available to carers.

Further objectives include:

- To determine the effects of COPe-support on carers’ mental wellbeing;
- To determine the effects of COPe-support on carers’ mental health knowledge, perceived support, appraisal of caregiving experiences, expressed emotion, and quality of life;
- To explore the intervention effects of COPe-support on hypothesized mechanisms of change through impacts on carers’ knowledge, perceived support, and appraisal of caregiving;
- To assess the relationship between usage of COPe-support and effectiveness in carers’ outcomes; and
- To determine the acceptability of COPe-support as perceived by carers.

#### Study hypotheses

The primary hypothesis is that COPe-support is superior to the attention-matched control with respect to improving carers’ mental wellbeing, measured using Warwick Edinburgh Mental Wellbeing Scale (WEMWBS [29]) at the end of intervention use of 20 weeks (primary end point). Secondly, it is hypothesized that relative to the control group, COPe-support participants will have better mental health knowledge, appraisal of their caregiving experiences, perceived support, and quality of life, at all follow-up time points.

## Methods

### Trial design

The study is an online randomized controlled trial (RCT) with an embedded internal pilot RCT to verify recruitment and retention; and a full RCT. The overall RCT uses a two-arm, individually randomized controlled superiority trial design comparing COPe-support (in addition to usual care) with an attention-matched control (in addition to usual care).

### Internal pilot

The trial includes a 12-month internal pilot to establish that study procedures work well to recruit and retain participants, and check that a good proportion of participants use the intervention. The definition of use is a participant who has activated their log-in and have accessed their allocated condition at least once. The internal pilot progression criteria include:

- **Go**: at least 80% of one-third of the sample size (i.e. 120) are recruited, and at least 80% of these participants have met the usage requirement and are retained in the study by 20 weeks (primary end point)
- **Amend and review**: If any of the recruitment, usage and retention rates are below 80%

We were required to achieve the above criteria by the end of the initial 12 months for the trial to proceed into the full study. In the event of any of the recruitment, retention, or usage thresholds not being met, we would have worked together with the Trial Steering Committee to review strategies for improvement. The internal pilot study was successful with all the “Go” criteria met prior to the deadline, and hence it proceeded into the next stage with further recruitment sites set up and recruitment strategies scaled up for a further year.

### Setting and participants

The study will recruit adult carers who provide ongoing care and support for a relative or close friend affected by psychosis (including Type 1 bipolar disorder in addition to all diagnoses covered by the NICE guideline on psychosis and schizophrenia [2]) on an unpaid basis. Both the carers and the cared-for individuals need to reside in England, UK.

#### Eligibility criteria

Inclusion criteria include family members, relatives or close friends who:

- Are aged 18 or over;
- Have at least weekly contacts with the cared-for person, in any form ranging from face-to-face to social media communications;
- Living in England during the study period;
- Able to communicate in English in usual online communications; and
- Have daily access to the internet including emails.

To avoid a clustering effect, we can only include one carer per cared-for individual in the trial. Therefore, we exclude potential participants who have another relative or friend, already enrolled in the study, sharing the caring role for the same person.

### Interventions

#### Active intervention: COPe-support

The intervention condition, COPe-support, is delivered through a web-based virtual learning environment (VLE) called Canvas (https://www.canvasvle.co.uk). The VLE allows the participants to access the intervention online via desktop or laptop web browsers, as well as smartphones or tablets through a Canvas app. COPe-support was co-produced through participatory research with a wide range of experts through experience or through professional training, building upon results identified through systematic reviews of the literature and a focus group study with carers and people affected by psychosis [6, 17, 22]. After the COPe-support prototype was finalized, we ran a mixed-method study to establish its feasibility and usability with a small sample of carers and technical expert. We also gleaned further usage and qualitative feedback from the usability study to refine the content and facilitation strategy of COPe-support [30]. These have been incorporated to develop and revise the delivery and facilitation strategies of the RCT, as reported in this protocol.

Delivered through an online enriched environment platform, COPe-support is a multi-component digital intervention providing psychoeducation and network support function with peers and experts. After logging in, carers will automatically enter into the Home page showing a brief introduction and a grid-based visualization representing the intervention menu [22, 30]. From here, carers can access and download a navigation video or equivalent textual guide. Using a mixture of textual and audio-visual materials devised by the study team with contributions from expert members, COPe-support content is grouped into 12 broad sections [22, 30]. These include:

- six sections of psychoeducation focusing on psychosis, its treatment, and related caring issues;
- two sections on wellbeing promotion information and exercises directed at carers themselves;
- two online forums, one called “Ask the Experts” where carers can post questions for advice from a panel of experts, and the other called “Peer to Peer” where carers exchange views with one another;
- a “Resources for carers” section with supplementary weblinks to a wide range of relevant external resources; and
- a “Support” section where participants can get in contact for technical or emotional support directly.

**Fig. 1:**
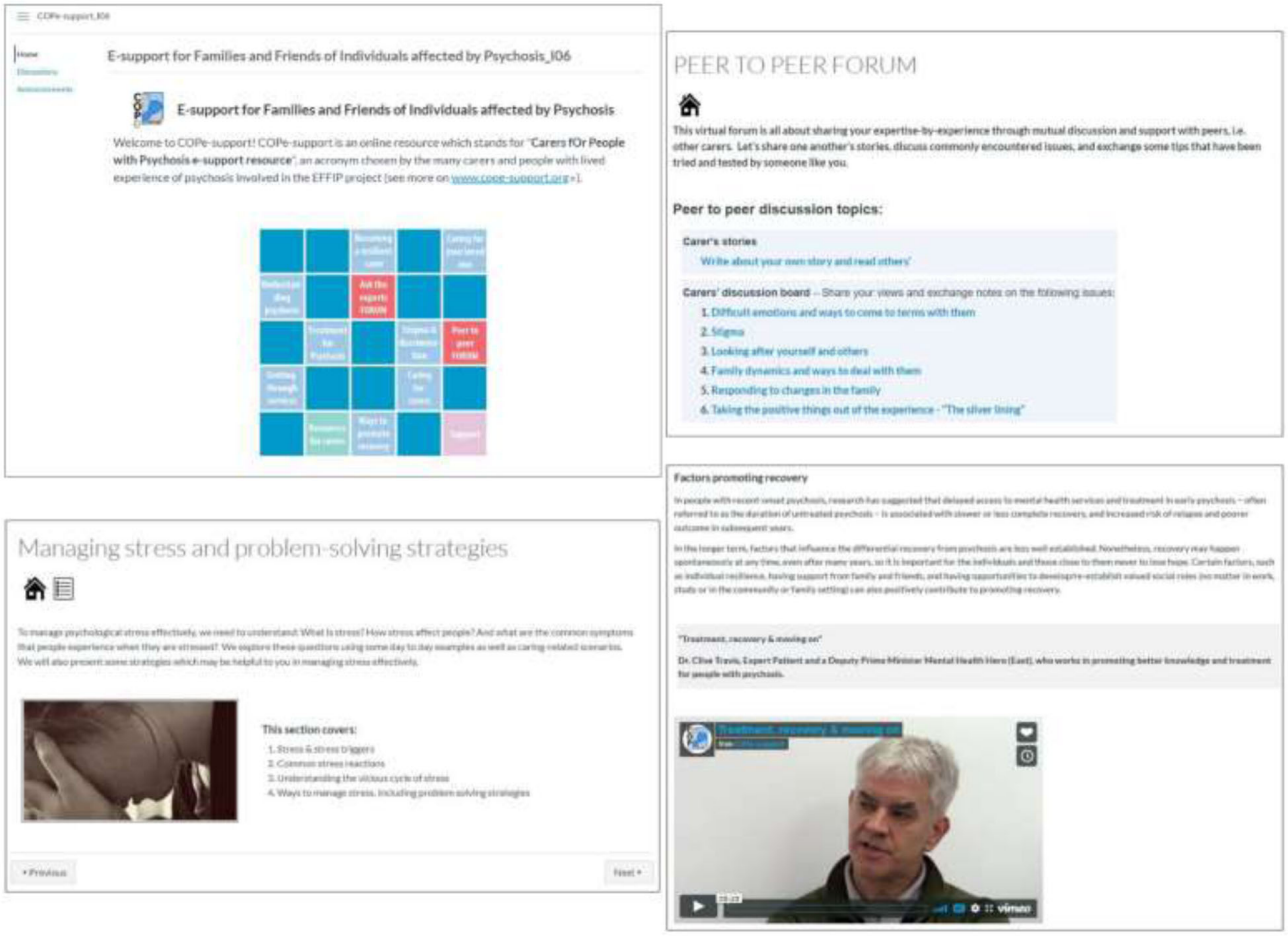
Screenshots of COPe-support content. Detailed legend: Screenshots taken from COPe-support intervention platform

The content of COPe-support is summarized in Table 1. Figure 1 provides screenshots of various sections of COPe-support.

**Table 1:** Summary of COPe-support content

Section title	Content
Understanding psychosis	An overview of psychosis including common symptoms and co-occurring problems, a bio-psychosocial explanatory model of possible causes, and prognosis.
Treatment of psychosis	Information on evidenced-based pharmacological, psychological, social, and alternative treatment for psychosis, with reference to the NICE guidelines.
Caring for your loved one	Information on caregiving, communication, and problem-solving strategies for a range of common symptoms and related issues, such as: supporting your loved one with paranoid beliefs; and working out a relapse prevention plan.
Getting through services	Information on the health, social and wider service systems and ways to navigating through them for support for their loved ones and the carers themselves.
Ways to promote recovery	It describes how carers can help support their loved ones to live their life to the full and gives information on a variety of support resources through the healthcare and wider social network.
Caring for carers	Focusing on carers themselves, we share tips from other carers with experiential expertise and the literature in ways to look after their own wellbeing, including interactive resources (such as podcasts) promoting wellbeing.
Stigma and discrimination	Information on stigma and discrimination commonly faced by individuals with psychosis and those close to them. We give information on related law and rights and suggest ways and resources which help deal with stigma and discrimination.
Becoming a resilient carer	This section helps carers to reflect and draw together relevant information and skills gained for their caring situation and for looking after their own wellbeing. It concludes with an interactive plan where carers can tailor-make their own “caring for carer” plan integrated with a wellbeing-promotion toolkit.
Peer to peer forum	A virtual forum and blog space for carers to share experiences and discuss commonly encountered issues. It has seven topics covering carer’s story and various topics such as difficult emotions and way to come to terms with them, and “taking the positive things out of the experience”.
Ask the experts forum	An interactive forum where carers can post questions to an expert panel. The forum is organized around six topics, including talking therapies and psychosocial interventions, and general and physical health issues. All other carers can also post responses and follow-up questions onto the forum.
Resources for carers *(The control comprises this section)*	A live information bank that covers a range of relevant services and resources with direct weblinks. Categories of resources include: voluntary organisations; relevant organization providing mental health information and support; statutory health and social care services; books; online resources; health research information; and money matters advice.
Support *(The control comprises this section)*	We provide a direct weblink in this section where carers can contact the study team for emotional or technical support. Information on alternative relevant support services, such as NHS 111 and Samaritan are provided here.

Throughout the study period, an online facilitator (JS) who is an experienced mental health nurse, monitors and moderates all the interactive functions of COPe-support. These include the forums and the inbuilt communication weblinks such as messages the participants can send through COPe-support platform asking for support or seeking information. The online facilitator also coordinates a panel of 14 expert panel members comprising psychiatrists, GPs, pharmacists, benefits advisors, CBT therapists, social worker, mental health nurse, and Expert through Experience, behind the operation of the “Ask the Experts” forum. Once a week, the facilitator also posts an update via the COPe-support announcement function to all participants with an aim to keep them engaged. In parallel to the announcement appearing on COPe-support platform, an email will automatically be generated to each of the participants. For security and confidentiality considerations, participants are required to follow a set of ground rules in using COPe-support [22]. These include the use of a self-chosen pseudonym to anonymise participation in communication and interactions online and adhering to the confidentiality principles so not to include any of their own and their cared-for individual’s person-identifiable details when making posts on either forum. We provide guidelines with examples to explain what constitute person-identifiable data and how participants can fully participate in the interactive forums without giving any such data away. If necessary, the facilitator can delete or edit posts made by participants and moderate any particular participants’ access to the forums [30].

#### Attention-matched control: web-based information bank

**Fig. 2:**
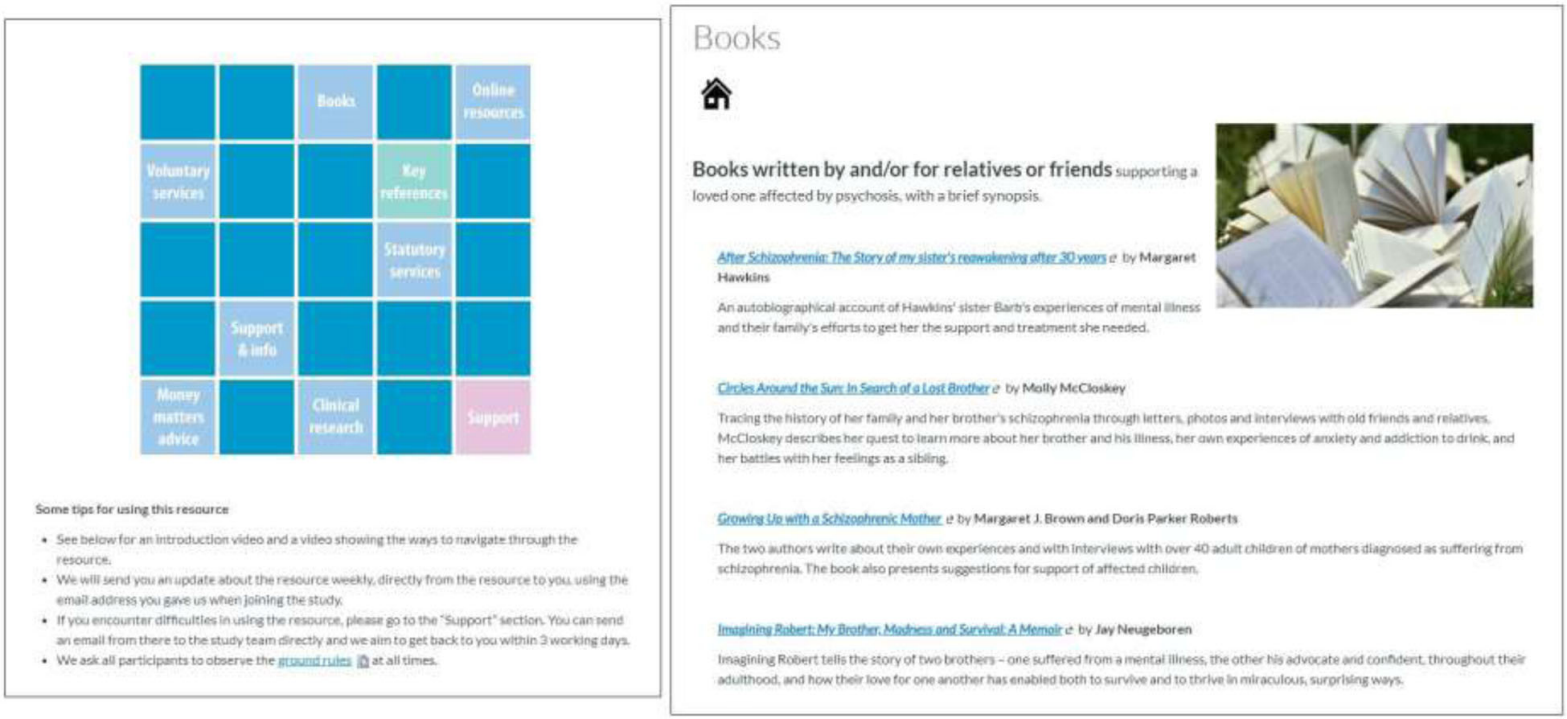
Screenshots of the control condition. Detailed legend: Screenshots taken from the control platform

To control for the attentional component of COPe-support and to account for the effects of online information provided by websites run by statutory and voluntary organisations as part of usual care, we developed a control intervention to have the identical look and “feel” as the active COPe-support intervention. We created the control condition mimicking the content of the Home page, “Resources for Carers”, and the Support section from the active intervention, also delivered through Canvas VLE (see Table 1). However, there are much less content and no interactive forums in the control nor any wellbeing promotion content. Participants can get in touch with the study team directly for support if necessary, and the control also has the same communication functionalities as the active intervention, hence participants will also be sent weekly updates. In order to encourage retention and follow-up data completion, we will offer the participants allocated to the control access to COPe-support for four months’ time, after final follow up data collection. Screenshots of the control are shown in Fig. 2.

### Procedures

#### Recruitment

We will recruit the participants from statutory mental health services across England, i.e. NHS mental health trusts which provide secondary mental health services locally, typically for a population between three-quarters of to one million. Furthermore, we will recruit through national and local voluntary mental health organisations (such as SANE http://www.sane.org.uk/ and Suffolk Family Carers https://suffolkfamilycarers.org/).

Study promotion and recruitment activities across all sites will include the research team presenting at relevant events targeting carers and staff working with carers. We will also disseminate flyers, posters, and study information widely within various sites, through working closely with the Clinical Research Network and local staff who are supporting the study recruitment. Social media advertising using twitter targeted at statutory and voluntary organisations which carers commonly seek out will also be utilized.

Further to the recruitment activities, all interested carers are directed to our study website (www.cope-support.org/) which provides comprehensive study information including the full participant information sheet outlining the study aims, risks and benefits, confidentiality and dissemination of results. Potential participants can register their interest in joining the study and undertake eligibility screening through the study website. Those who meet all the eligibility criteria are then guided through the online informed consent seeking and study enrolment process. The study website also provides direct weblink for potential participants to post any queries and get in touch with the study team.

**Fig. 3:**
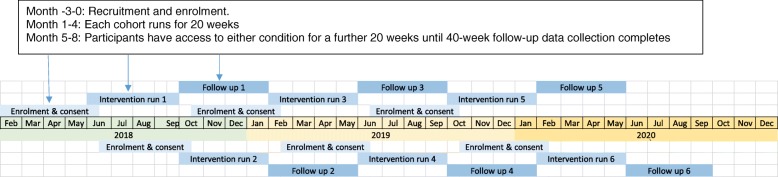
Schedule of cohorts. Detailed legend: Not applicable

**Fig. 4:**
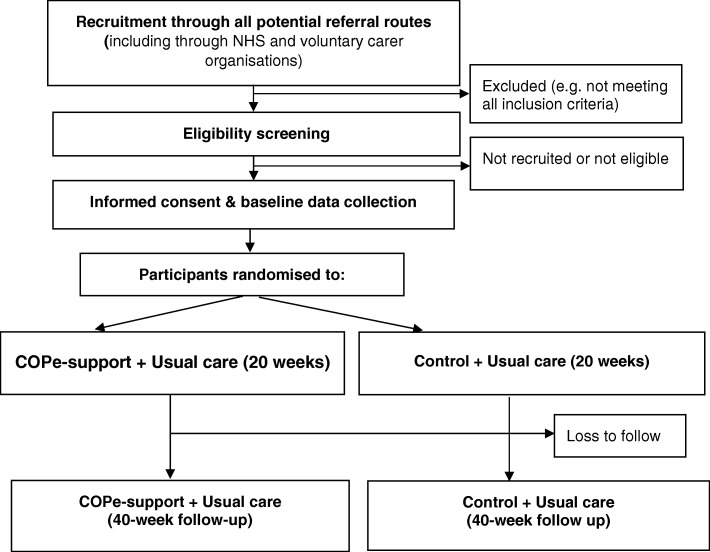
CONSORT flowchart showing participant pathway through the trial. Detailed legend: Not applicable

We schedule all the participants recruited over a duration of two years (i.e. March 2018 to February 2020) into six cohorts, each lasting eight months, as shown in Fig. 3. This approach allows us to group at least 20 but no more than 60 participants in the intervention platform at any one time. This is to ensure that forum elements of the intervention - those requiring interactions between participants – are accessed by an optimal number of participants [17]. Two weeks prior to each cohort is due to start, consented participants are invited to undertake a baseline measures through our secure online platform. Those who have completed the baseline measures will then be randomized to receive either COPe-support or the attention-matched control. They will be sent log-in details to access the respective condition and instructions to download the free app (Apple or Android) for those who want to use COPe-support (or the control) on tablets or smart phones, in addition to computers. The flow of participants through the study phases is shown in Fig. 4.

### Assessment

#### Primary outcome and measure

The primary outcome is carers’ mental wellbeing at 20 weeks assessed using Warwick Edinburgh Mental Wellbeing Scale (WEMWBS [29]). WEMWBS is a self-report measure comprising 14 short statements focusing on positive wellbeing. Each statement is rated on a 5-point Likert scale, resulting in the possible scores range from 14 to 70 (higher score indicates better mental wellbeing). Since its validation, WEMWBS has been widely used in epidemiological studies, including the Health Surveys in England and Scotland [11, 31, 32]. It has been established that an increase of 3 points in WEMWBS is a meaningful change post intervention, based on a wide range of mental health intervention trials undertaken in different populations [13, 33].

#### Secondary outcomes and measures

A range of secondary outcomes will be considered, including changes in the following:

Carers’ mental health knowledge, measured using the Mental Health Knowledge Schedule (MAKS [34]). MAKS has two sections: the former to investigate participants’ knowledge of mental health; the latter for contextualising the responses. Possible scores range from 6 to 30 by summing up the scores of the first section of six items. Higher scores of MAKS indicate better level of mental health knowledge.

Carers’ appraisal of caregiving experience, assessed using the Experience of Caregiving Inventory (ECI [8]). There are 66 brief statements each requiring a rating on a 5-point ordinal scale (from 0 = never to 4 = nearly always) in the ECI. These are categorized into ten subscales; eight negative subscales summing into the total negative appraisal total (possible scores range from 0 to 208; higher scores indicate poorer outcome), and two positive subscales totaling into the positive appraisal score (possible scores range from 0 to 56; higher the scores better the positive perspective).

Carers’ wellbeing and perceived support, measured with the Carers’ Wellbeing and Support Questionnaire (CWS [35]). CWS contains two subscales: wellbeing (higher scores indicate better wellbeing) and perceived support (higher scores indicated lower satisfaction).

Criticism and emotional over-involvement in the family, assessed by Family Questionnaire (FQ [36]). FQ comprises 20 short questions, with 10 of these covering the criticism subscale, and the remainders the emotional over-involvement subscale. Higher scores indicate poorer outcomes on both subscales.

Carers’ quality of life assessed using Euro-QoL 5-level (EQ-5D-5L [37]). EQ-5D-5L comprises two components: health state description covering the domains of mobility, self-care, usual activity, pain/discomfort and anxiety/depression; and an overall evaluation using a visual analogue score (EQ-VAS). The EQ-VAS asks the participants to rate their subjective rating of health ranging from 0 to 100, where 100 implies the best health one can imagine, and 0 the worst.

#### Service use

In addition to these measures, service use information by the participants will be collected to describe usual care.

#### Process evaluation outcomes and data

The trial has an inbuilt process evaluation study to explore participants’ usage patterns and subjective experience of participants in using COPe-support. In order to understand when and how participants access and use the online intervention, we will collect usage data which the intervention and control platform automatically monitors and records. These include:

- Number of log-ins on separate days per participant (both arms)
- Average page views per log-in per participant (both arms)
- Total time spent on the platform per participant (both arms)
- Average time spent per page view on the platform per participants (both arms)
- Total number of posts per participant made to the peer-to-peer forum (intervention arm only)
- Total number of posts per participant made to the Ask the Experts forum (intervention arm only)

Perceived acceptability will be determined through interviewing a purposive sample of 20% of the participants in the intervention arms of the trial after all the data collection procedures (i.e. about 36 out of the 180, or less should data saturation, that is no further data is being collected that adds to the content of existing themes, be reached in the iterative analysis process [38]). For the individual interview, purposive sampling will be used to identify participants to ensure representation of diverse experiences and views from those with ethnic minority backgrounds, different caregiving experiences and roles, and usage patterns. The interview is designed to be semi-structured and conducted over phone or Skype, suiting the participants’ preference and convenience.

### Data collection

Assessments will be completed online at baseline (i.e. no more than two weeks prior to randomization and allocation), half-way through the intervention use (10-week), post intervention (20-weeks), and 40-week follow-up. All outcome measures are self-completed questionnaires as described above. At each time point, we will invite the participants to undertake an online assessment through our secure platform. At study enrolment, we will also collect data on participant’s demographic and caregivng-related information (e.g. the gender of the cared-for person, are they living together or separately, and how much time they spend on caring for their loved one in the previous week). At each assessment time point, participants will receive up to six email-reminders over a four-week window, linking them to an online platform through which they can complete the assessment.

We will collect the usage data during the intervention time (20 weeks) following completion of all outcome data collection, i.e. at the end of 40 weeks. These data will be collected from our intervention (and control) platform which automatically record individuals’ usage.

**Fig. 5:**
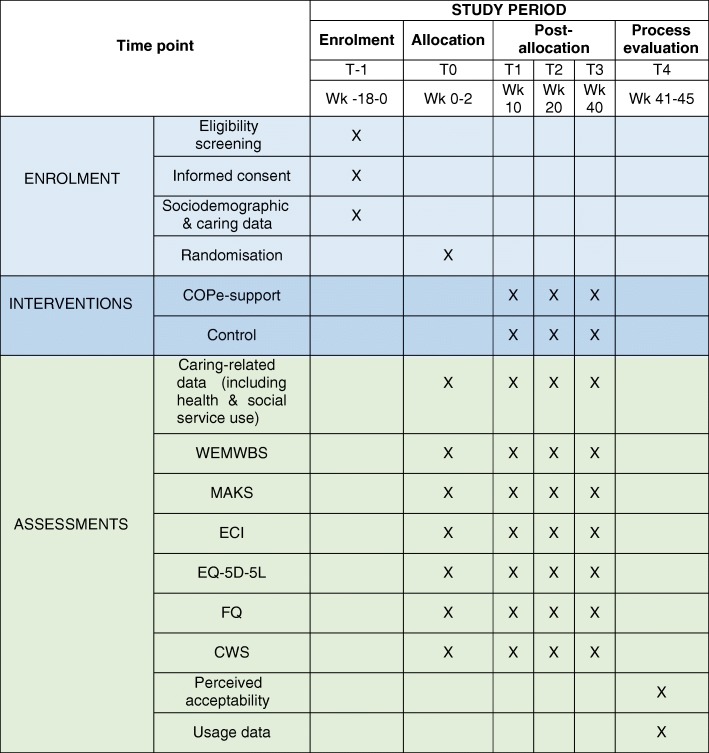
Schedule of data collection. Detailed legend: T-1: Pre-randomisation; T0: baseline and randomisation; T1: mid-intervention; T2: end of intervention; T3: 40-week follow up; T4: after follow-up outcome data collection; WEMWBS: Warwick-Edinburgh Mental Wellbeing Scale; MAKS: Mental Health Knowledge Schedule; ECI: Experience of Caregiving Inventory; EQ-5D-5 L: EuroQol 5 level version of EQ-5D; FQ: Family Questionnaire; CWS: Carer Wellbeing & Support Questionnaire

On completion of each assessment, participants will be emailed an Amazon voucher to thank for their time and participation (i.e. £10 for the first, £5 for the second and third respectively, and £10 for the fourth and last). We will offer an additional £10 Amazon voucher for those who give an interview. Figure 5 shows all the measures used and data collection schedule.

### Sample size

The trial sample size is calculated with reference to an earlier study testing a digital health intervention targeting siblings of individuals with first episode psychosis [39] and the primary outcome measure data (i.e. WEMWBS [29]). This study is powered to detect the minimum clinically important difference of + 3 in WEMWBS which is considered a meaningful change post intervention between treatment groups [31]. Using 80% power, significance level of 5% and an estimated SD of 9 from the E Sibling Project RCT baseline results [13, 39], 143 carers per arm are needed. With estimated 20% attrition, additional participants (*n* = 74), will be recruited, leading to an overall sample size of 360 across both arms.

### Random allocation

Participants who have completed baseline measures before the respective cohort starts will be randomized using a 1:1 ratio to “COPe-support and usual care” versus “Control and usual care” by an online randomization programme developed and hosted by King’s Clinical Trials Unit. The algorithm for randomization will consist of permuted blocks stratified by the gender. While there is no established evidence of any carer sociodemographic or caregiving-related factors having an effect on carers’ health outcomes, females (having a caregiving role or not) are known to have worse outcomes in population mental wellbeing research [11, 12, 40] and that participants in most prior research recruiting carers were predominantly females.

### Allocation concealment and blinding

We are unable to blind participants to their allocation as they will realise that the control condition has much less content, but both arms will have access to an online resource bank and usual care. Nonetheless, all outcome data collected use self-reported measures and direct online input by participants. The study coordinator and intervention (and control) online platform facilitator (JS) cannot be blinded to the allocation because she needs to adjust her facilitation according to the different content of the two groups.

### Data storage and security

All trial data as described above are collected through an online system linked and integrated with the intervention platform. Only the Chief Investigation (JS) and core study team members who have a role in either study facilitation or data management will have direct access to such platform and data. All data sets will be password protected. To ensure confidentiality, data dispersed to trial statisticians and wider project team members will be anonymized of any identifying participant information.

### Monitoring

#### Trial steering committee

The trial is overseen by an independent Trial Steering Committee (TSC) comprising clinical-academic experts, an independent trials statistician, and carers with experiential expertise on the subject. The TSC provides governance and guidance on the study delivery and progress. It provides a monitoring and advice function for the safety and wellbeing of the study participants. In view of the safety event data, the TSC will consider the need for appointing a separate Data Monitoring Committee (DMC) if necessary. In addition, the TSC reviewed and approved the safety protocol, usage data definitions and statistical analysis plan.

### *Safety protocol*

For this trial we have adapted the definition of adverse events commonly used in conventional clinical trials.

In addition to adverse and serious adverse events, we have devised a category labelled as “unintended consequences”. These are summarized as follows:

- Unintended consequences (UCo) – incidents during which participants’ use of the intervention (or control) is interrupted and the potential distress caused is below the threshold of that described in adverse event category. Examples include participants forgetting their log-in password, platform technical issues interrupting delivering, or participants realizing their own unmet needs in light of the intervention content.
- Adverse events (AE) - clear evidence of distress or concerns of risk of harm towards participants or others, resulting in needs for medical and/or mental health support. However, there is no immediate or serious threat of severe harm or risk of life
- Serious adverse events (SAE) – clear evidence of immediate risk to life or welfare for the participants or others.

Monitoring of all potential safety concerns will cover both active monitoring of the online platform communications (e.g. participants’ post made on forums) and query/concerns raised by participants (e.g. those who send messages through our platform). In all circumstances, the online facilitator will contact the participant concerned and offer support through online or phone communication, in addition to signposting them to relevant local services. In the unlikely event of SAEs, we will contact the relevant local authorities (e.g. Police or social services) as appropriate. Such safety monitoring and support mechanisms are outlined in the participant information sheet.

Recording and coding of safety and untoward events will be conducted on a continuous basis. These will also be reported to the TSC prior to each of the biannual TSC meeting or at an ad hoc basis if indicated, both for consensus on coding and for safety monitoring. In the event of SAEs, these need to be reported to both the TSC chair and the sponsor within 48 hours.

Overall adverse effects, including the number of UCo, AE, and SAE, will be recorded by number of events, types and participants across both arms. In addition, we will also record and report participant withdrawals from the study.

### Trial status and review

The trial is registered with the Current Controlled Trials registration under the title “Randomised controlled trial of COPe-support online resource for carers” (ISRCTN 89563420 [10.1186/ISRCTN89563420], registration date: 02/03/2018). This study has also been reviewed and approved by South Central – Oxford C Research Ethics Committee (Reference: 18/SC/0104) and Health Research Authority (Reference: IRAS 240005). Recruitment for the trial commenced in March 2018 and will continue till early 2020. Participants are covered by indemnity and insurance for harm associated with the protocol provided by the Sponsor, St George’s, University of London.

### Data analysis

#### Statistical analysis plan

The data analysis will be performed by the trial statisticians who will be blind to randomized allocation for the primary and secondary outcome analysis. Primary analyses will be undertaken on an intent-to treat (ITT) basis, including all participants who were randomized and supplied at least one post-randomisation measure at 10, 20, or 40 weeks, regardless of usage status or withdrawal from the study. For the primary analysis, a linear mixed model will be used. The model will include participant and cohort as random effects and arm, time (10, 20, 40 weeks), the randomization stratification variable gender as well as baseline score and a time-by-arm interaction. In order to improve precision we will adjust for variables parent (Y/N) and living with the cared-for individual (Y/N). The model’s assumptions for random effects distributions and residuals will be investigated. If assumptions are not appropriate then transformation will be considered. This model will be used to estimate the mean difference and 95% CI in WEMWBS between arms at the 20-week time point (i.e. primary endpoint) though we will also explore and report the intervention effect at week 10 and 40. The model will be fitted using restricted maximum likelihood (REML). Secondary outcome measures will be assessed using similar approach. No interim analyses will be performed.

It is unlikely that missing baseline data will be problematic for the analysis as baseline outcomes are required to be collected prior to randomization. Further, as the analysis uses a mixed model which is efficient for missing data under the missing at random assumption [41] no multiple imputation will be performed. Sensitivity analysis will include exploring the mean WEMWBS score at each time point if all participants with missing information was set at the highest and lowest values seen in the dataset.

Further analysis after the analyses of primary and secondary outcomes will not be blind as this is not feasible. The intervention effect in those that use COPe-support (defined as having at least two logins) will be estimated using a complier adjusted case analysis (CACE) with “usage” treated as both a binary and ordinal variable.

#### Mediation measures analysis

A mediation measures analysis will be performed if there is an intervention effect identified through the primary analysis. We will explore if the effect worked through the mechanisms underlying the intervention design and as hypothesized by the adapted stress-appraisal-coping model applied in family caregiving [22, 42]. The mechanisms to be investigated include:

- Appraisal measured by ECI [8] – exploring the effect of cognitive perception of the caregiving situation as the stressor;
- Perceived support measured using the support subscale of CWS [35] – via use of the peer-to-peer forum and satisfaction with support received;
- Mental health knowledge assessed by MAKS [34] – via psychoeducation provided by the intervention.

The mediation analysis will allow us to decompose the total observed intervention effect into mediated (indirect) and non-mediated (direct) component and we will use a structural equation modelling approach. Wellbeing (WEMWBS) will be measured at 20 weeks and 40 weeks and mediators at all time points (i.e. 10, 20 and 40 weeks) but “adjusted” for previous level and/or baseline as indicative. We will fit each mediator in turn separately and then undertake a multiple mediation analysis. A full statistical analysis plan will be available on request from the lead trial statistician (VC).

#### Qualitative data analysis

Transcribed qualitative data collected on participants’ experience in using COPe-support will be analysed using the framework analysis method [38, 43], in order to explore participants’ experiences and understanding of the processes whereby the intervention might bring about change.

### Reporting and dissemination

The trial will be reported following the Consolidated Standards of Reporting Trials guideline [44]. We aim to disseminate the results of this study widely, through publication in peer-reviewed journals and presentations at national and international conferences. Our study website (www.cope-support.org) will serve as a communication portal providing updates and outputs from the study and links to all publications.

## Discussion

Despite clinical guidelines recommending psychoeducation, especially those delivered to carers using a group format as an effective support for carers, its provision remains scarce [2, 16]. Caregiving, although bringing positives to both personal experiences and bonds with significant others, often causes burden and distress in carers. Given carers’ wellbeing is correlated with their caregiving capacity, which can also affect the quality of support the cared-for person receives, it is paramount that carers have access to an effective treatment. Digital mental health interventions delivered through the internet offer novel and flexible solutions to disseminating evidence-based psychosocial interventions to carers [17]. The trial of COPe-support represents one step closer to providing carers access to digital mental health interventions.

This study will provide valuable evidence regarding the effectiveness of a web-based psychoeducation intervention integrated with peer support for carers. Despite the proliferation of digital mental health interventions, there is scarce research on the effectiveness in the field of psychosis [17]. Meanwhile, carers’ need for such an intervention maybe particularly high given their diverse needs and many other commitments, such as work and their own families. Digital interventions offer advantages in that they increase user autonomy and anonymity, which may be important as stigma and lack of knowledge of, or access to services can impact upon help-seeking behaviour in carers [17, 45]. An intervention delivered through the internet may also offer the only means to provide, through the healthcare system, highly individualised care to a critical mass of carers [46].

While optimising all the potentials the internet offers into the study and intervention design, we are aware of the challenges common amongst digital interventions and online trials. Because digital interventions allow their users a self-directed approach in using the content, intervention usage or adherence is often much more heterogenous [23, 47]. Trial attrition and disengagement are also expected issues [23, 25]. To account for potential drop out and disengagement, we have devised a facilitation protocol alongside the delivery of COPe-support, drawing strategies and ideas through extensive consultations with carers and a usability study on the intervention-prototype [22, 30]. We have used conservative recruitment and retention estimation taking lessons from the few other similar trials with carer populations [39, 48]. Furthermore, we have also used a trial design that includes an internal pilot with keen monitoring on these parameters. We will review and revise our strategies at end of pilot to scale up to the full trial accordingly. In terms of the usage issue, we have put in place several strategies. We recognise COPe-support is a novel intervention delivered through the internet, and as such a conventional “adherence threshold” or per protocol usage pattern would not be appropriate. As no literature-based usage was identified [6], we consulted experts through experience and clinical and academic experts in the field to identify and define the meaningful usage definitions used in the study protocol. We are particularly interested in understanding (1) how carers will use COPe-support, and (2) if there is a differential intervention effects in those who have decided to log-in COPe-support twice or more and use it in different extent. For such goals, we consider it important to carry out the “complier average causal effect” (CACE) analysis [23, 27]. This is because the primary analysis using the ITT model, although the gold standard, will have neglected the usage effects, if any.

To our knowledge, the current study is one of the first to test online psychoeducation integrated with peer support for psychosis carers, compared to an attention control. In addition to enhance participant retention, we are mindful to design the control to match online resources typically available to carers as part of usual care. Although the choice of an attention control may reduce effect sizes, this stricter examination should result in stronger arguments for the use of interactive and moderated web-based interventions (such as COPe-support), rather than non-interactive information passively delivered through the internet.

## Data Availability

The datasets generated during and/or analysed during the current study will not be shared or publicly available due to conditions on participant consent and other ethical restrictions, as registration of the trial predated open data recommendations.

## Abbreviations

AE: Adverse event
CACE: Complier adjusted case analysis
COPe-support: Carers fOr People with Psychosis e-support
CWS: Carer Wellbeing & Support Questionnaire
ECI: Experience of Caregiving Inventory
EFFIP: E-support for Families and Friends of Individuals affected by Psychosis Project
EQ-5D-5L: EuroQol 5 level version of EQ-5D
FQ: Family Questionnaire
ITT: Intention-to-treat
MAKS: Mental Health Knowledge Schedule
RCT: Randomized controlled trial
SAE: Serious adverse event
TSC: Trial Steering Committee
UCo: Unintended consequence
UC: Usual care
VLE: Virtual learning environment
WEMWBS: Warwick-Edinburgh Mental Wellbeing Scale
